# Investigation of protists in Momoge wetland (China) through metagenomic next-generation sequencing

**DOI:** 10.3897/BDJ.13.e153721

**Published:** 2025-07-03

**Authors:** Yuting Huang

**Affiliations:** 1 College of Life Science, Baicheng Normal University, Baicheng, China College of Life Science, Baicheng Normal University Baicheng China

**Keywords:** protist, biodiversity, Momoge wetland, metagenome

## Abstract

The Momoge wetland plays an important role in maintaining the ecosphere and protist is an indispensable component of it. In order to reveal community structure and diversity of protists in Momoge wetland, metagenomic next-generation sequencing (mNGS) was performed. The main results are as follows: 1) A total of 224 species were identified, belonging to 17 phyla, 32 classes, 75 orders, 94 families and 146 genera. Among them, Bacillariophyta, Evosea, Oomycota, Rhodophyta, Ciliophora, Haptophyta, and *Salpingoecarosetta*, *Guillardiatheta*, *Polarellaglacialis*, *Cladocopiumgoreaui* were the dominant phyla and species, respectively; 2) Most of them were species adapted to the saline-alkali environment, and the protists of Momoge wetland had higher diversity, fewer dominant species, and higher evenness than those of the harsher environment; 3) KEGG analysis showed that some protistan pathways were related to the saline-alkali environmental adaptation. This research is beneficial to ecological protection and provides valuable information for future studies.

## Introduction

The Momoge wetland is located in Zhenlai County, Baicheng City, Jilin Province, China, and its altitude ranges from 130 to 145 m a.s.l. The climate type is temperate continental monsoon, with an average annual temperature of 4.2 ℃ and an average annual precipitation of 392 mm (Cui et al. 2021). It is a typical saline-alkali wetland (Liu et al. 2022). There are numerous lakes, which are an important habitat for migratory populations of white cranes. It is a significant international wetland, which was also officially listed in The Ramsar Convention’s List of Wetlands of International Importance in October 2013 (Cui et al. 2021). The protist is a main link for the transfer of matter (such as carbon and energy of food webs), which sustains primary productivity in aquatic ecosystems (Meira et al. 2018, Cruaud et al. 2019). However, there is little research on protists of the Momoge wetland.

In recent years, metagenomic next-generation sequencing (mNGS) has been employed to provide a comprehensive view of protists in wetlands. For instance, the protistan communities in wetlands were studied to elucidate the dominant species and examine the diversity (Liu et al. 2024, Ogola et al. 2024). Based on mNGS technology, the present study conducted a survey on the composition of protists in Momoge wetland, as well as an analysis of KEGG pathways, aiming to provide information of their diversity and functions.

## Material and methods

**Sampling and sequencing**

**Figure 1.**
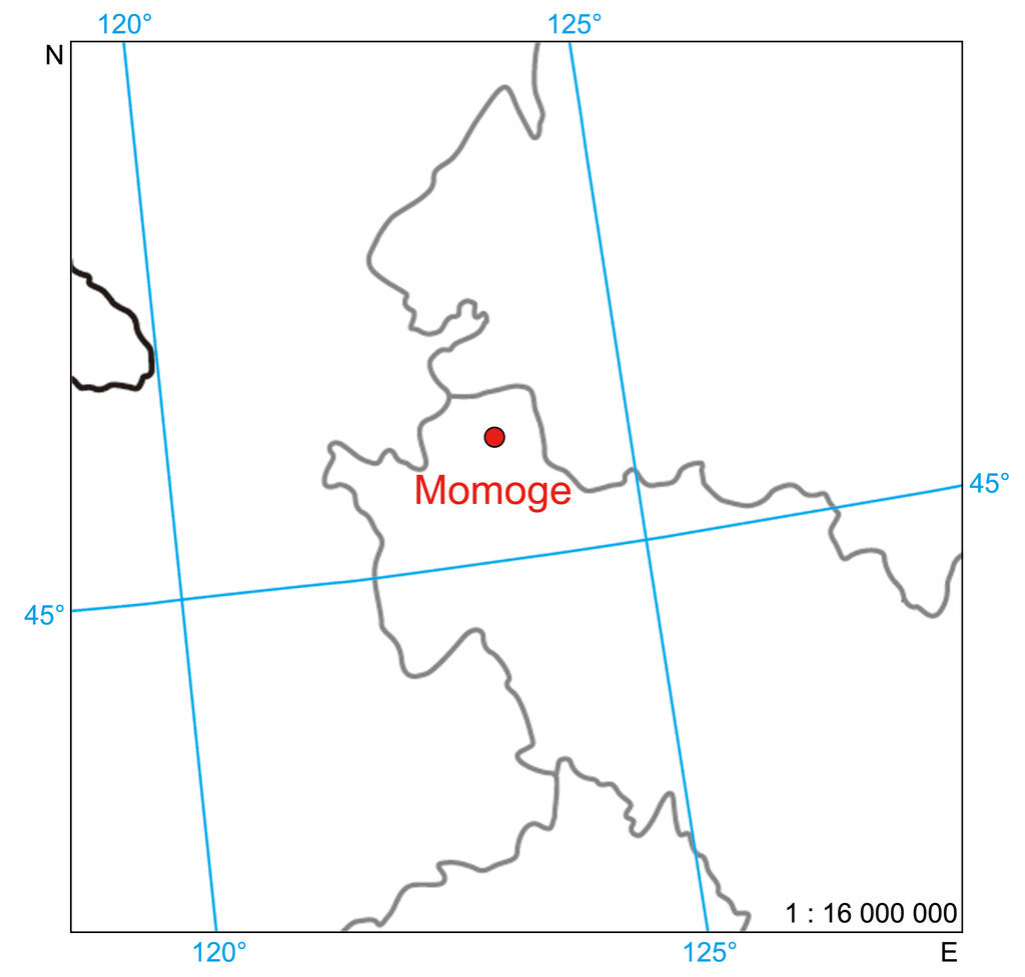
The geographic coordinate of the sampling site.

Three parallel water samples were collected from Momoge wetland (45°93′N, 123°56′E) (Figure 1) on August, 2024 and filtered through 0.22 μm polycarbonate membranes (Taoyuan, CN). DNA was extracted using Mag-Bind Soil DNA Kit (Omega Bio-tek, USA), and integrity was detected by 1% agarose gel (Thermo, USA). The concentration was measured by Quantus Fluorometer (Promega, USA). Then DNA was fragmented into 350bp by Covaris M220 (Covaris, USA), and NEXTFLEX Rapid DNA-Seq (Bioo Scientific, USA) was used to construct libraries. Illumina NovaSeq 6000 (Illumina, USA) was employed for sequencing in China.Fig. 1

**Analysis of data**

FASTP v0.20.0 was used for low-quality bases (Q-score≤20) removing (Chen et al. 2018). The reads after quality control were assembled by using MEGAHIT v1.1.2 (Li et al. 2015). CD-HIT v4.6.1 was adopted to cluster the coding sequences to construct the non-redundant gene set (Fu et al. 2012). SOAP v2.21 was used to compare reads with the non-redundant gene set to obtain the abundance information (Li et al. 2008). The sequences of non-redundant gene set were compared to the Non-Redundant Protein Sequence database by using DIAMOND v2.0.13 for species annotation (Buchfink et al. 2021). The package “vegan” of R v4.3.2 was used to calculate the indices of diversity including Shannon, Simpson, and Pielou's evenness with default parameters (R Core Team 2023). GhostKOALA v3.1 were adopted for KEGG annotation, which were assigned to specific species based on their genes (Kanehisa et al. 2016) and the network diagram was drawn with Gephi v0.10.1 (Bastian et al. 2009).

## Results

**The sequencing results**

The total raw and clean data of mNGS were 97.0 and 95.3 Gb, respectively. The results of assembly are shown in Table 1. The raw data have been deposited into the NCBI Sequence Read Archive with BioProject accession number PRJNA1230694.

**The composition and diversity of protists**

**Figure 2.**
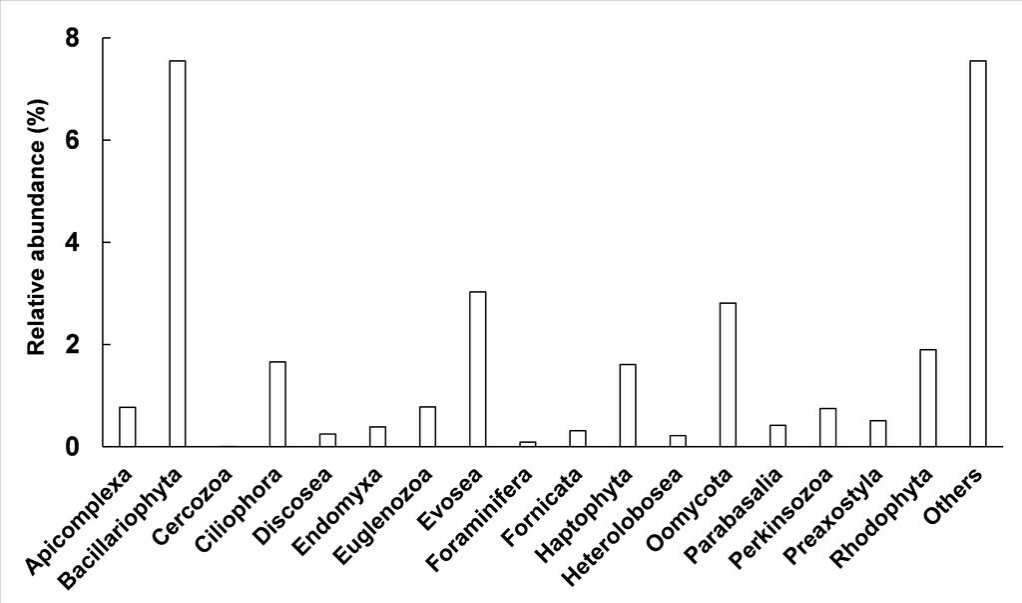
The composition of protists in Momoge wetland. The percentages of relative abundance are at phylum level.

**Figure 3.**
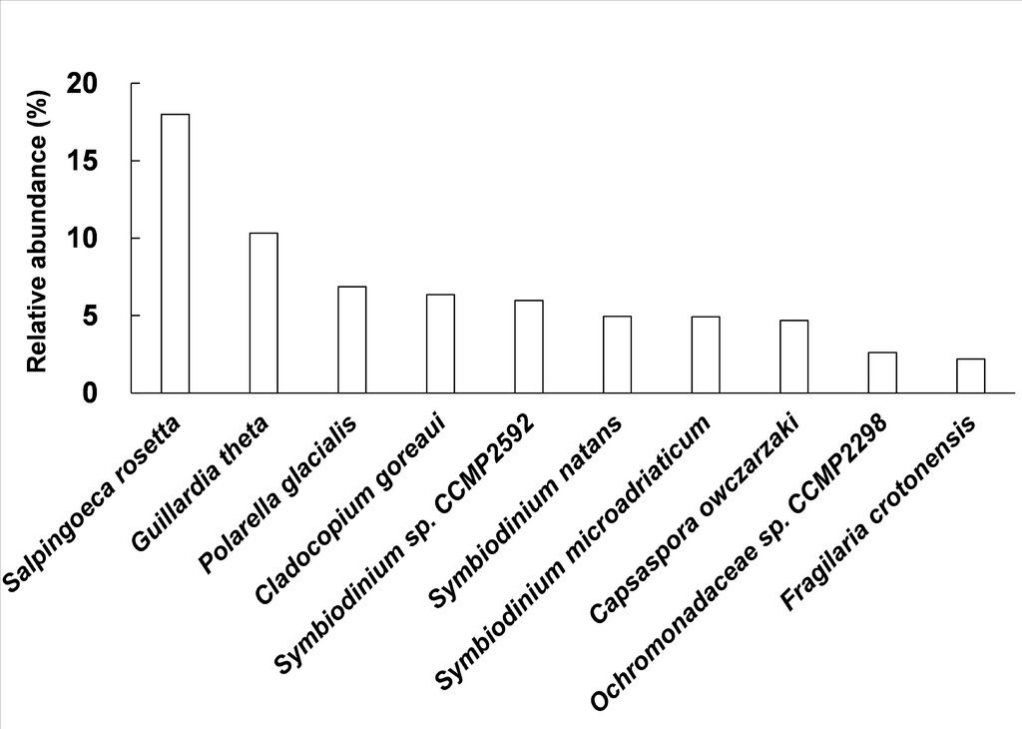
The percentages of the top ten species ranked by relative abundance.

A total of 17 phyla of protists were identified, corresponding to 32 classes, 75 orders, 94 families, 146 genera, and 224 species (Fig. 2, Suppl. material 1). In the protist community of Momoge wetland, Bacillariophyta (7.55%), Evosea (3.03%), Oomycota (2.81%), Rhodophyta (1.90%), Ciliophora (1.66%), and Haptophyta (1.61%) were the dominant phyla determined by relative abundance, except for others that could not be classified at this level (Fig. 2). As shown in Fig. 3, the percentages of the top species ranked by relative abundance were *Salpingoecarosetta* (17.99%), *Guillardiatheta* (10.32%), *Polarellaglacialis* (6.86%), *Cladocopiumgoreaui* (6.35%), *Symbiodinium* sp. CCMP2592 (5.98%), *Symbiodiniumnatans* (4.95%), *Symbiodiniummicroadriaticum* (4.92%), *Capsasporaowczarzaki* (4.68%), *Ochromonadaceae* sp. CCMP2298 (2.61%), and *Fragilariacrotonensis* (2.19%).

**Figure 4.**
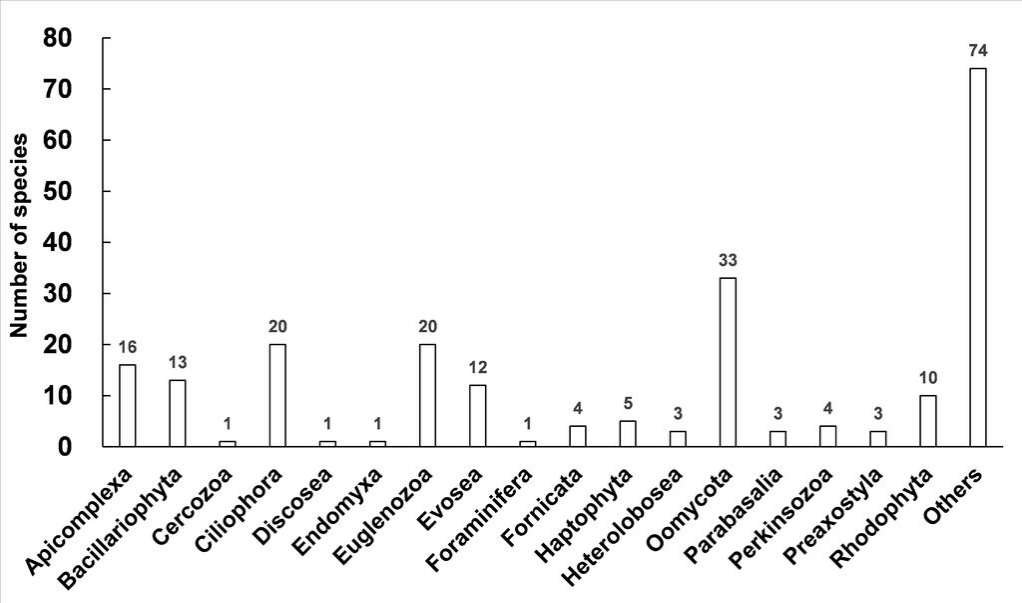
The numbers of species in phyla.

The numbers of species in phyla were shown in Fig. 4 and the species richness of Oomycota, Ciliophora, Euglenozoa, Apicomplexa, Bacillariophyta, Evosea, Rhodophyta, and Haptophyta was higher. The Simpson and Shannon indices were 0.07 and 3.49, respectively (Table 2). In addition, the diversity indices at phylum level are also listed in Table 2. The Oomycota, Euglenozoa, Ciliophora, Apicomplexa, Evosea, Bacillariophyta, Rhodophyta and Haptophyta had higher Shannon indices, while Parabasalia, Perkinsozoa, Rhodophyta, Preaxostyla, Heterolobosea, Fornicata had higher Simpson indices (Table 2). The Pielou's evenness of Heterolobosea was the highest and that of Rhodophyta was the lowest (Table 2).

**The KEGG network of protists in Momoge wetland**

**Figure 5.**
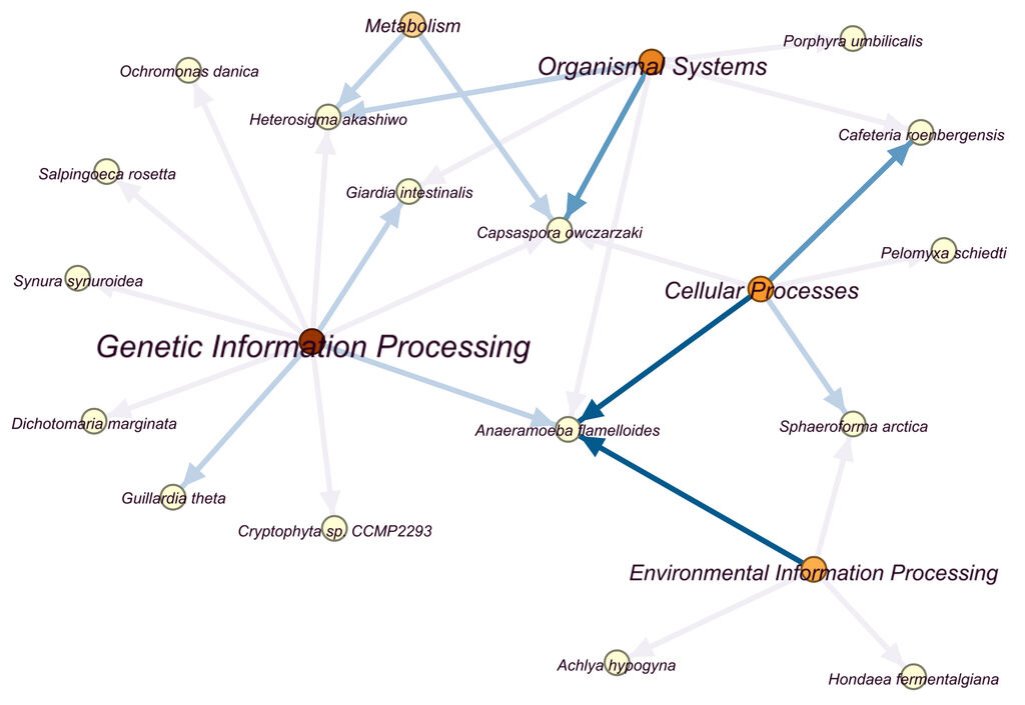
The KEGG network of protists in Momoge wetland. The red and orange nodes: pathway categories; yellow nodes: protists; edges: relations between protists and pathways categories.

As shown in Fig. 5, the darker the color of pathway category nodes, the greater the number of species involved, and the darker the color of edges, the greater the number of pathways that species participated in under this category. The most annotated genes of species participated in genetic information processing, followed by organismal systems, cellular processes, environmental information processing, and metabolism. The *Anaeramoebaflamelloides* was closely related to cellular processes and environmental information processing. Besides *A.flamelloides*, *G.theta* and *Giardiaintestinalis* were highly involved in genetic information processing. Both organismal systems and metabolism were highly associated with *Capsasporaowczarzaki* and *Heterosigmaakashiwo*. The details of KEGG pathways are listed in Suppl. material 2.

## Discussion

**The community and diversity of protists**

The mNGS was performed (Table 1) and Bacillariophyta, Evosea, Oomycota, Rhodophyta, Ciliophora, and Haptophyta were the dominant phyla of the protistan community in Momoge wetland (Fig. 2). The percentages of the dominant species and the diversity indices are also displayed in Fig. 3 and Table 2, respectively. Momoge wetland is a typical saline-alkali wetland (Liu et al. 2022). Diatoms are common components of saline wetlands (Colla et al. 2022), and bacillariophyta (diatom) is the dominant group of autotrophic algae in brackish waters (Mann et al. 2016), which is similar to the result of this study (Figs 2, 4). It has also been found in other saline-alkali wetlands (Zhao et al. 2023). Both *Porphyridiumpurpureum* and *Galdieriasulphuraria* have the characteristic of salt tolerance (Lu et al. 2020, Abiusi et al. 2021). In this study, they had the higher relative abundance within the Rhodophyta (red algae) (Suppl. material 1). The species richness of Rhodophyta was also high (Fig. 4). Ciliates are dominant components of hypersaline habitats (Harding and Simpson 2018, Weinisch et al. 2019) and both the proportion and species richness of Ciliophora were also relatively high in Fig. 2 and Fig. 4. It is worth noting that *Pseudocohnilembuspersalinus*, which is a halophilic ciliate (Weinisch et al. 2019), had the highest relative abundance within the Ciliophora (Suppl. material 1). Although Heterolobosea is also halophilic (Harding and Simpson 2018), its relative abundance was not high in this study (Fig. 2), and the reason needs to be investigated. As for Haptophyta (Suppl. material 1), *Emilianiahuxleyi* tolerates a broad range of salinity conditions (Sheward et al. 2024). *S.rosetta*, as the top specie in Momoge wetland (Fig. 3), has close relatives that are highly adapted to the hypersaline environment (Schiwitza et al. 2018). *Guillardia* of Cryptophyta has high relative abundance in salt lake (Yang et al. 2024). In this study, the relative abundance of *G.theta* within the same genus was also high (Fig. 3). It has been reported that filasterean *C.owczarzaki*, which has high relative abundance as shown in Fig. 3, can respond to osmotic stress conditions (Shabardina et al. 2023). These results in the present study indicate that most of the dominant protists were those adapted to saline-alkali environment. In addition, both Evosea and Oomycetes can degrade organisms (Thines 2018, Bosch et al. 2024). Their high relative abundances and species richness (Figs 2, 4) as well as the high Shannon index of Oomycetes (Table 2) suggest that they were main consumers of saprophytic nutrients in Momoge wetland. The Shannon, Simpson, and Pielou's indices measure the species diversity, dominance, and evenness of a community, respectively (Shannon 1948, Simpson 1949, Pielou 1966). In the present study, the Shannon index, Simpson index, and Pielou's evenness of protists were 3.49, 0.07, and 0.65 (Table 2), while these indices are 2.93, 0.15, and 0.56 in Tibetan Plateau’s wetland (Zhang et al. 2022). It can be seen that compared to those of Tibetan Plateau’s wetland, protists of Momoge wetland had higher diversity, fewer dominant species, and higher evenness. This might be due to the harsher environment of the plateau. Moreover, the dominant phyla also demonstrated higher Shannon indices generally, which means that they were the main contributors to the total value of Shannon (Fig. 2,Table 2). However, the values of Simpson and Pielou's evenness (Fig. 2,Table 2) suggested that the composition of species in some dominant ones was not as uniform as that in others.

**The KEGG functions of protists**

The network between species and KEGG pathways was constructed (Fig. 5, Suppl. material 2). As seen from the graph, the *A.flamelloides* was closely connected with cellular processes and environmental information processing. It is an anaerobic organism (Åberg 2024), and there are currently no reports that it tolerates saline-alkali conditions. According to Suppl. material 2, it participated in KEGG pathways such as cellular senescence, FoxO signaling pathway, PI3K-Akt signaling pathway, Wnt signaling pathway, Hedgehog signaling pathway, Hippo signaling pathway, JAK-STAT signaling pathway. These pathways mainly maintain normal physiological functions such as development (Rawlings et al. 2004, Evangelista et al. 2006, Pan 2010, Jafari et al. 2019, Hayat et al. 2022). In addition, the relations between genetic information processing, organismal systems, metabolism and *G.theta*, *G.intestinalis*, *C.owczarzaki*, as well as *H.akashiwo*, were also close (Fig. 5). As mentioned above, *Guillardia* has been found in salt lake (Yang et al. 2024) and the osmotolerance contractile vacuole is also in *G.theta* (Hoef-Emden 2014). Its KEGG pathways of genetic information processing were protein processing in endoplasmic reticulum and basal transcription factors (Suppl. material 2). Whether these pathways are related to the formation of contractile vacuoles requires further verification. The KEGG pathways of filasterean *C.owczarzaki* were glycosylphosphatidylinositol (GPI)-anchor biosynthesis, SNARE interactions in vesicular transport, endocytosis and so on (Suppl. material 2). It has been reported that phosphatidylinositol signaling, SNARE proteins, endocytosis are related to the salt tolerance of eukaryotes (Salinas-Cornejo et al. 2021, Salinas-Cornejo et al. 2023, Yang et al. 2024). As for *H.akashiwo*, its main KEGG pathways were oxidative phosphorylation, thermogenesis, and retrograde endocannabinoid signaling (Suppl. material 2). Except for that, the heterotrophic nanoflagellates *Cafeteriaroenbergensis* (De Corte et al. 2019) also participated in cellular processes quite a lot (Fig. 5) and its main KEGG pathways included apoptosis, antigen processing and presentation, autophagy, lysosome, and phagosome (Suppl. material 2). Similarly, these pathways were identified in the KEGG analysis of other eukaryotic organisms under saline-alkali environments (Shi et al. 2023, Jin et al. 2024, Wang et al. 2025).

## Conclusions

In summary, this research accomplished investigation of protists in Momoge wetland. Based on mNGS technology, the dominant phyla and species were identified and the diversity indices were also calculated. The analyses revealed the KEGG functions of protists as well. In detail, the Bacillariophyta, Evosea, Oomycota, Rhodophyta, Ciliophora, Haptophyta, *S.rosetta*, *G.theta* , *P.glacialis*, and *C.goreaui* were the dominant ones in Momoge wetland and most of them are adapted to the saline-alkali environment. The protists in Momoge wetland had higher diversity, fewer dominant species, and higher evenness than those in harsher environment. Some KEGG pathways of *A.flamelloides*, *G.theta*, *G.intestinalis*, *C.owczarzaki*, *H.akashiwo*, and *C.roenbergensis* were involved in normal physiology, while some were related to the saline-alkali environment’s adaptation. As shown in the present study, compared to traditional approaches, the mNGS is culture-free and it can obtain functional annotations. The information regarding protists in Momoge wetland will be beneficial for the maintenance and protection of ecological diversity, as well as future studies.

## Supplementary Material

BDF21D91-EB4F-56C4-BAF0-CA053CE5B640 10.3897/BDJ.13.e153721.suppl1 15812319 Supplementary material 1 Table S1 Data type table Brief description The classification of protists in Momoge wetland File: oo_1279365.xlsx https://binary.pensoft.net/file/1279365 Yuting Huang

811BC245-755F-513C-B104-47952C399C3A 10.3897/BDJ.13.e153721.suppl2 15812321 Supplementary material 2 Table S2 Data type table Brief description KEGG pathways of protists in Momoge wetland File: oo_1350508.xlsx https://binary.pensoft.net/file/1350508 Yuting Huang

## Figures and Tables

**Table 1.** Summary of assembly.

**Sample Name**	**Contigs Number**	**Contigs bases (bp)**	**N50 (bp)**	**N90 (bp)**
Momoge_1	2,690,889	1,449,649,057	523	331
Momoge_2	2,724,190	1,454,135,895	525	331
Momoge_3	2,748,576	1,659,312,158	617	342

**Table 2.** Diversity indices of protists in Momoge wetland.

**Index**	**All**	**Apicomplexa**	**Bacillariophyta**	**Ciliophora**	**Euglenozoa**	**Evosea**	**Fornicata**	**Haptophyta**	**Heterolobosea**	**Oomycota**	**Parabasalia**	**Perkinsozoa**	**Preaxostyla**	**Rhodophyta**
Shannon index	3.49	2.00	1.98	2.07	1.70	1.98	0.95	1.25	0.94	2.82	0.69	0.78	0.83	1.06
Simpson index	0.07	0.19	0.17	0.18	0.36	0.16	0.42	0.32	0.43	0.09	0.61	0.58	0.52	0.54
Pielou's evenness	0.65	0.72	0.77	0.69	0.57	0.80	0.68	0.78	0.86	0.81	0.63	0.56	0.75	0.46
